# Oscillatory characteristics of the visual mismatch negativity: what evoked potentials aren't telling us

**DOI:** 10.3389/fnhum.2013.00426

**Published:** 2013-08-01

**Authors:** George Stothart, Nina Kazanina

**Affiliations:** School of Experimental Psychology, Faculty of Science, University of BristolBristol, UK

**Keywords:** mismatch negativity (MMN), visual attention, theta oscillations, alpha oscillations, phase locking, evoked potentials, induced oscillations

## Abstract

The visual mismatch negativity (vMMN) response is typically examined by subtracting the average response to a deviant stimulus from the response to the standard. This approach, however, can omit a critical element of the neural response, i.e., the non-phase-locked (“induced”) oscillatory activity. Recent investigations of the oscillatory characteristics of the auditory mismatch negativity (aMMN) identified a crucial role for theta phase locking and power. Oscillatory characteristics of the vMMN from 39 healthy young adults were investigated in order to establish whether theta phase locking plays a similar role in the vMMN response. We explored changes in phase locking, overall post-stimulus spectral power as well as non-phase-locked spectral power compared to baseline (−300 to 0 ms). These were calculated in the frequency range of 4–50 Hz and analysed using a non-parametric cluster based analysis. vMMN was found intermittently in a broad time interval 133–584 ms post-stimulus and was associated with an early increase in theta phase locking (75–175 ms post-stimulus) that was not accompanied by an increase in theta power. Theta phase locking in the absence of an increase in theta power has been associated with the distribution and flow of information between spatially disparate neural locations. Additionally, in the 450–600 ms post-stimulus interval, deviant stimuli yielded a stronger decrease in non-phase-locked alpha power than standard stimuli, potentially reflecting a shift in attentional resources following the detection of change. The examination of oscillatory activity is crucial to the comprehensive analysis of a neural response to a stimulus, and when combined with evoked potentials (EPs) provide a more complete picture of neurocognitive processing.

## Introduction

Mismatch negativity (MMN) is an electrophysiological response that reflects the automatic detection of change in the sensory environment, and is elicited by violating an established regularity in a sequence of sensory stimuli. Such violations can take the form of simple physical changes in the stimulus properties, e.g., a change in pitch of an acoustic stimulus (Paavilainen et al., 1993), to abstract deviations in the relationships between stimuli, e.g., missing a step in a musical scale (Brattico et al., 2006), or a non-symmetrical stimulus in a sequence of symmetrical stimuli (Kecskés-Kovács et al., 2012). Since its first description (Näätänen et al., 1978; Näätänen and Michie, 1979) it has become an established tool in the investigation of sensory processing and attention, and a marker of cognitive decline across a variety of conditions (see Näätänen et al., 2011 for a review). After the initial focus on the auditory MMN (aMMN), there is now an established body of evidence for MMN in the visual modality, the vMMN (see Pazo-Alvarez et al., 2003; Kimura et al., 2011; Winkler and Czigler, 2012 for reviews).

The typical method for measuring the MMN response is to subtract the evoked potential (EP) response to the standard repeating stimulus from that of the deviant stimulus, i.e., the stimulus that violates the regularity established by the standard. The resulting difference wave reflects the neural processing difference between the standard and deviant stimuli. Statistical techniques vary but typically the aim is then to establish the duration and magnitude of any deviation in the difference wave from zero.

In addition to examining electrophysiological responses with amplitude as a function of time, the same responses can also be examined in the frequency domain, in order to examine the oscillatory characteristics of a response as a function of time. Since the first observation of event related oscillatory changes (Berger, 1929), oscillatory activity has been increasingly shown to play a key role in exploring sensory, cognitive and motor processes (see Başar et al., 2001; Ward, 2003; Buzsáki and Draguhn, 2004 for reviews). Typically oscillations are separated into the following bands for analysis, delta (0–4 Hz), theta (4–8 Hz), alpha (8–14 Hz), beta (14–30 Hz), and gamma (30 + Hz). In the visual modality, processes that are involved with or influenced by the vMMN response have been associated with different oscillatory processes. Distracter suppression and selective attention processes have been linked with alpha oscillation changes (Foxe and Snyder, 2011), object feature binding (Gray et al., 1989; Tallon-Baudry and Bertrand, 1999) and visual working memory (Tallon-Baudry et al., 1998; Rizzuto et al., 2003) have been associated with increases in gamma and theta oscillations. Mishra et al. (2012) identified concomitant increases in theta phase locking and power as oscillatory markers of visuo-spatial attention.

It should be noted that a stimulus can elicit both stimulus phase-locked (sometimes known as “evoked”) and non-phase-locked (sometimes known as “induced”) oscillatory changes, however it is only the phase-locked activity that will sum to form the characteristic peaks and troughs of a recognizable EP in the time domain. Non-phase-locked activity, because its phase does not align from trial-to-trial, will fail to sum to any meaningful activity in an averaged EP in the time domain. Non-phase-locked oscillatory activity has been suggested to play an important role in the synchronization and desynchronization of functional networks in the brain (Bastiaansen et al., 2012).

Recently the oscillatory characteristics of the aMMN have been examined using time-frequency analyses. Fuentemilla et al. (2008) demonstrated that the frontal and temporal sources of the aMMN were differentially modulated by stimulus phase-locked theta power increase and theta phase locking. The frontal source of the aMMN showed an increase in stimulus phase-locked theta power following deviant stimuli and an increase in phase locking. The temporal sources however showed an increase in theta phase locking in the absence of any increase in power. In a magnetoencephalographic (MEG) study of aMMN, Hsiao et al. (2009) demonstrated partially converging results with Fuentemilla et al., specifically an increase in theta phase locking and power in response to deviant stimuli at temporal sources, and an increase in theta phase locking at frontal sources only. Changes in power at the frontal sources were not reported so it is unclear if findings on the frontal sources matched those of Fuentemilla et al. Furthermore, Hsiao's study demonstrated increases in power and phase locking to deviant stimuli that were greater in the right hemisphere than the left. Ko et al. (2012) also demonstrated that aMMN was associated with increases in both theta power and phase locking, peaking at fronto-central electrodes and stronger on the right hemisphere. Bishop and Hardiman (2010) examined the aMMN response in single trials using principle components analyses and also found a significant increase in theta phase locking. Although there are differences in the results and in analyses techniques between studies (e.g., electrode sites chosen for analyses), a clear role for theta oscillatory activity in the aMMN response has emerged, with a trend for right hemispheric dominance.

The present study investigated the role of neural oscillations in the vMMN response. By examining visual evoked potentials (VEPs), stimulus phase-locked and non-phase-locked spectral power change, and inter trial phase locking (ITPL) across a range of frequencies (4–50 Hz), we were able to examine whether theta activity plays a similar role in the generation of the vMMN response as has been demonstrated in aMMN.

## Materials and methods

### Participants

Thirty-nine healthy younger adults [aged 18–31, mean age 20.0 (± 2.3), 13 males] gave consent to participate in the study. Participants were recruited from the University of Bristol student population and declared themselves to be in normal health. All had normal or corrected-to-normal vision and were right hand dominant [mean Edinburgh Handedness Inventory score 94.4 (± 14.4)]. Seventeen of the younger adults were control participants in a previously reported study examining vMMN in healthy ageing (Stothart et al., 2013), however the study only examined vMMN in the classic time-amplitude domain and no time-frequency analyses have been previously reported. All appropriate approvals for our procedures were obtained from the Ethics Committee of the Faculty of Science at the University of Bristol. Participants provided written informed consent before participating and were free to withdraw at any time.

### Stimuli

Stimuli were presented using Presentation software version 12.2 (Neurobehavioral Systems, Inc).

### Procedure

**Figure 1:**
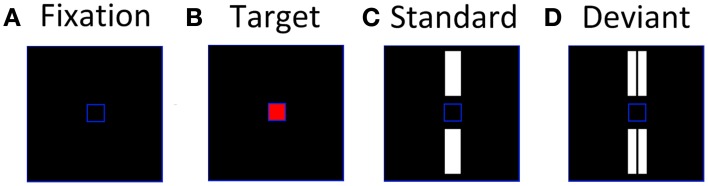
**Stimuli used to elicit visual evoked potentials, (A) inter-stimulus screen; (B) attended target; (C) unattended standard; (D) unattended deviant**.

Using a paradigm previously developed by Tales et al. (1999), participants were instructed to fixate and attend exclusively to a small blue frame (1.3 × 1.3 cm) at the center of a monitor situated 0.5 m directly in front of them (Figure 1A). Periodically, the center of the blue frame turned red (the target stimulus) (Figure 1B) and the participant had to respond to it as quickly as possible by pressing a hand-held button. Participants were instructed to ignore any other stimuli that appeared on screen and focus solely on the target stimuli. The target presentation was a rare event for which subjects would have to maintain a sharp attentional focus, thereby reducing the likelihood of attending to the standards and deviants. A larger blue frame (10.5 × 10.5 cm) defined the area within which the standard and deviant stimuli were presented. The standards, single white bars (3.9 × 1.2 cm) were presented simultaneously above and below the central blue square (Figure 1C); deviants, double white bars equal to the standards in total area (3.9 × 0.6 cm ×2) and brightness, were presented in the same locations (Figure 1D). The symmetrical location of standards and deviants about the target area was intended to minimize any tendency for gaze fixation to be biased away from the central square. The target, standard and deviant stimuli were presented with a randomized inter-stimulus interval (ISI) of 612–642 ms for 200 ms. Furthermore, the targets and deviants were presented in a pseudo-random sequence among the standards with at least two standards preceding each deviant. The ratio of standards:deviants:targets was 16:1:1. Standards and deviants were not counterbalanced as it has been previously demonstrated using this paradigm that it is the rareness of the deviant rather than the subtle difference in stimulus characteristics that elicits the vMMN response (Stagg et al., 2004). The stimuli were shown in one block lasting 11 min containing 640 standards, 40 deviants, and 40 targets.

### EEG recording

EEG signals were continuously recorded from 64 Ag/AgCl electrodes fitted on an elasticized cap in a standard electrode layout using a common FCz reference. Signals were sampled at a rate of 1000 Hz using a BrainAmp DC amplifier (Brain Products GmbH). Impedances were kept below 5 kΩ and all signals were online low-pass filtered at 250 Hz during recording. Recordings were analysed offline using Brain Electrical Source Analysis software version 5.3 (BESA GmbH). Artifacts including blinks and eye movements were corrected using BESA automatic artifact correction (Berg and Scherg, 1994) and any remaining epochs containing artifact signals > ±100 μV were rejected. The rejection rate never exceeded 10% of trials for each participant and condition. Data were re-referenced offline to a common average reference. Epochs from −300 to 600 ms were defined around stimulus onset and baseline corrected using the pre-stimulus interval (−300 to 0 ms).

### ERP analysis

To confirm the presence of a vMMN the amplitudes of seven electrodes, O1,Oz,O2,PO9,PO10,PO7, and PO8, were averaged to form an occipital region of interest. Electrode selection was defined based on a recent study of vMMN using an identical paradigm (Stothart et al., 2013). Examination of grand average evoked responses and mean spectral power maps across the scalp confirmed that the overwhelming majority of neural activity was located in the occipital region, was highly consistent across the seven electrodes, and that the electrode selection was appropriate. The averaged response to the standard stimuli was subtracted from that to the deviant stimuli to create a difference waveform and a 40 Hz low-pass filter applied (only for the VEP analysis, not applied for frequency analysis). Sequential one sample *t*-tests were then applied to the difference waveforms for each group using the method outlined by Guthrie and Buchwald (1991). The consecutive time points necessary to indicate an epoch of significant difference between the standard and deviants responses were obtained from a simulation using an autocorrelation estimated from the data. Time intervals with values of *p* < 0.05 that lasted for the required duration, 15 consecutive time points (i.e., 15 ms), were accepted as significantly different epochs.

### Time-frequency analyses

In order to further characterize the vMMN response, original epochs (i.e., not subjected to any offline filtering) from all 64 electrodes were transformed into the time-frequency domain using a complex demodulation approach, implemented by BESA Source Coherence module version 5.3 (Hoechstetter et al., 2004). Complex demodulation was applied using a sampling step of 2 Hz for frequencies between 4 and 50 Hz and a finite impulse response filter with a sampling step of 25 ms at latencies between −300 to 600 ms relative to the stimulus onset. Changes in spectral power were calculated relative to pre-stimulus baseline (−300 to 0 ms) in two different ways. First, demodulation of all activity (stimulus phase-locked and non-phase-locked) was performed by calculating spectral content of each epoch on a trial-by-trial basis and then averaging it (“overall spectral power”). In order to specifically assess non-stimulus phased locked activity, we first subtracted the participant's average response in the time-frequency domain from each individual trial, and then averaged the trials to create averages of the non-phase-locked spectral power only. ITPL values (i.e., the degree to which oscillatory phase is correlated from trial to trial, ranging from 0 to 1, with values approaching 1 indicating highly correlated phase values) were calculated for the overall spectral activity. For the analysis of phase locking values the number of standard trials was matched to the deviant trials by selecting the standard preceding the deviant for analysis. This equated signal to noise ratios between standard and deviant stimuli and removed the potential influence of the number of presentations on correlation values.

### Statistical analysis

All time frequency data were analysed using a non-parametric cluster based permutation approach. This approach, using Fieldtrip software (Oostenveld et al., 2011), and described by Maris and Oostenveld (2007), controls for multiple comparison testing when computing statistics across multiple frequency and time points. Firstly an independent samples *t*-test between the standard and deviant conditions was calculated for each sample point. Significant values (alpha < 0.01) were clustered based on their adjacency in time, space and frequency, and the *t*-values for all points in this cluster were summed. The critical *p*-value for each cluster was calculated using the Monte Carlo estimate. For each cluster this involved randomly dividing the data into two subsets and calculating a new summed *t*-value. This was repeated 10,000 times and the proportion of random partitions that resulted in a larger summed *t*-value than the one observed in the real data identified. If the summed *t*-value of the observed data cluster was higher than 95% of the random partitions (i.e., less than an alpha-level of 0.05, two-tailed), then the cluster was considered to represent a significant difference between the two groups. This technique allows for the evolution of spectral activity across time to be observed without the need for reductive averaging across arbitrary time windows, grouping of frequencies into bands or imposing spatial constraints on cluster size. It should be noted that the initial alpha value for cluster formation was lowered from alpha < 0.05 to alpha < 0.01 in order to reduce the likelihood of large clusters spanning the entire dataset, a potential problem in cluster based permutation testing highlighted recently by Mensen and Khatami (2013).

For the purposes of effective visualization time frequency data is presented for the averaged activity across the seven electrodes used in the ERP analyses (i.e., O1,Oz,O2,PO9,PO10,PO7, and PO8). Grand average and statistical plots based on the cluster based permutation analysis for all 64 electrodes are available in Supplementary Figure A.

## Results

### VEP

**Figure 2:**
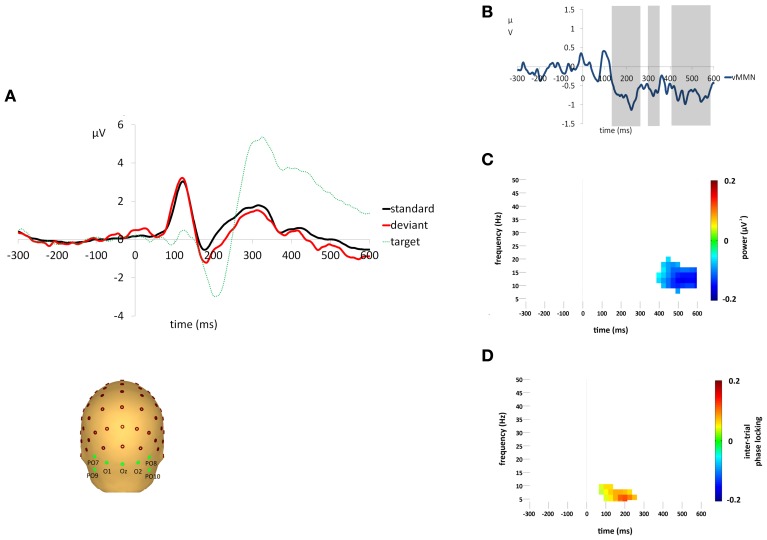
**(A)** Grand average responses to standard, deviant and target stimuli measured at the occipital region of interest. Electrodes O1,Oz,O2,PO9,PO10,PO7, and PO8 were averaged to form the occipital region of interest for VEP analyses. **(B)** Difference waveforms (i.e., deviant minus standard) illustrating the vMMN response. Shaded areas indicate epochs of significant difference (*p* < 0.05) between responses to standard and deviant stimuli. **(C)** Difference (i.e., deviant minus standard) in non-phase-locked spectral power change (μV^2^) compared to baseline (−300 to 0 ms) for the average activity across the occipital region of interest. Plot shows the significantly greater reduction in alpha power (*p* < 0.05) for responses to deviant stimuli after Monte Carlo permutation correction for multiple comparisons, non-significant differences are masked in white. **(D)** Difference (i.e., deviant minus standard) in ITPL value (ranging from 0 to 1, with values approaching 1 indicating highly correlated phase values) compared to baseline (−300 to 0 ms) for the average activity across the occipital region of interest. Plot shows the significantly higher ITPL value (*p* < 0.05) for responses to deviant stimuli after Monte Carlo permutation correction for multiple comparisons, non-significant differences are masked in white.

A clear vMMN response was observed, see Figures 2A,B. Sequential *t*-tests corrected for multiple comparisons identified three epochs (133–263 ms, 297–352 ms, 377–584 ms) in which deviant responses were significantly more negative than standards. Target stimuli elicited clear attentional components, i.e., P3b, that was not present in either the standard or deviant stimuli. The mean percentage of targets detected was 98.4% (±0.02) and the mean median reaction time was 390.8 ms (±48.4). There were no false alarm responses to any deviant stimuli for any participants.

### Changes in overall (phase-locked and non-phase-locked) spectral power

**Figure 3:**
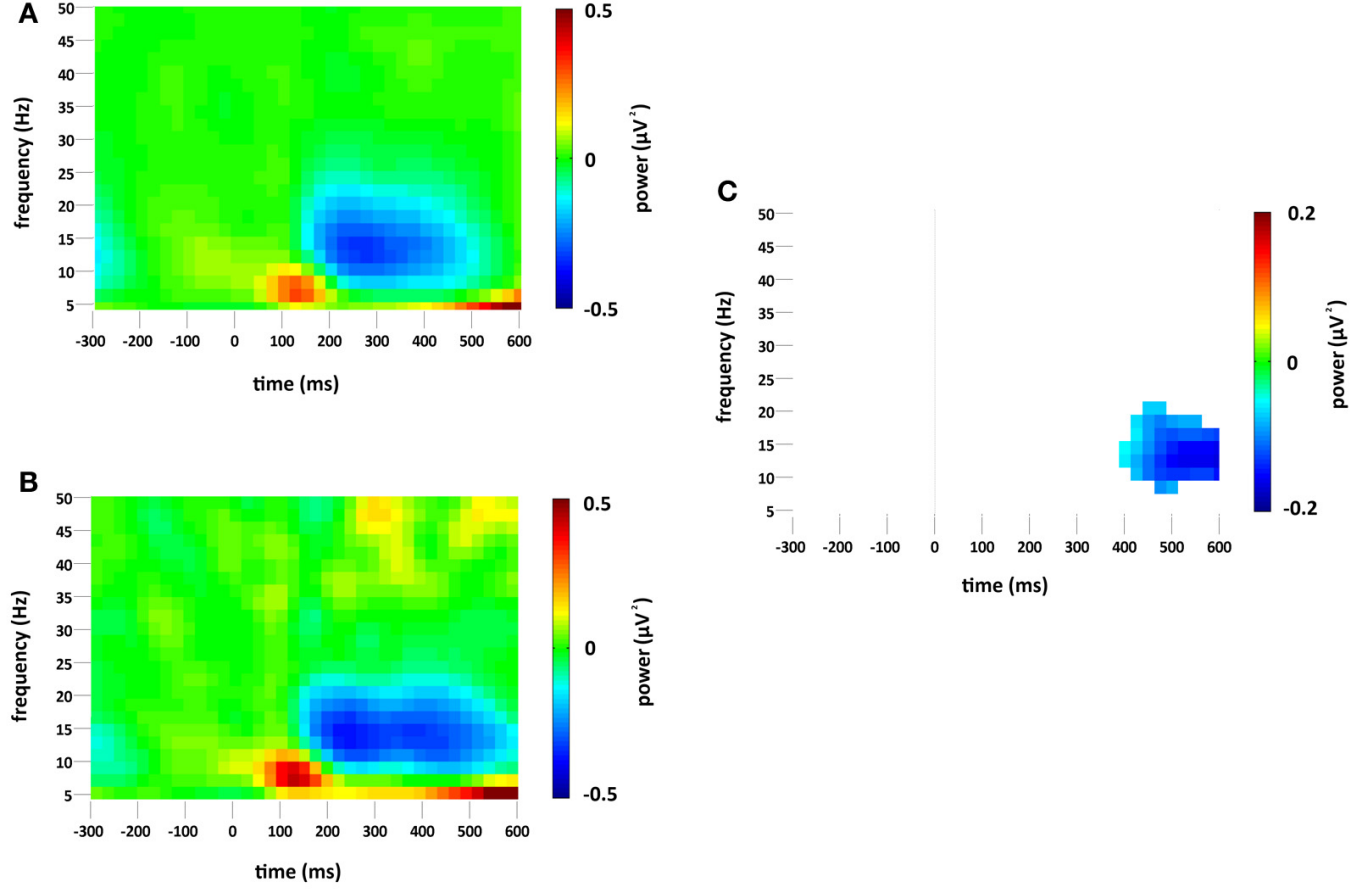
**Overall (i.e., stimulus phase-locked and non-phase-locked) spectral power change (μV^2^) at the occipital region of interest compared to baseline (−300 to 0 ms) for (A) standard and (B) deviant stimuli.** Plot **(C)** significant difference values (i.e., deviant minus standard, *p* < 0.05) after Monte Carlo permutation correction for multiple comparisons. Non-significant differences are masked in white.

**Figure 4:**
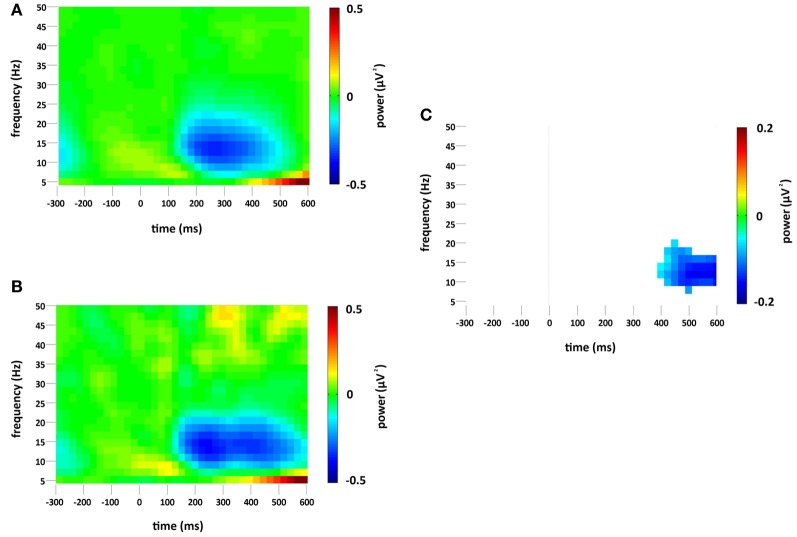
**Non-phase-locked spectral power change (μV^2^) at the occipital region of interest compared to baseline (−300 to 0 ms) for (A) standard and (B) deviant stimuli.** Plot **(C)** significant difference values (i.e., deviant minus standard, *p* < 0.05) after Monte Carlo permutation correction for multiple comparisons. Non-significant differences are masked in white.

Standard and deviant stimuli elicited an increase in the overall spectral power, greatest at 6 Hz, between approximately 75 and 175 ms (see Figures 3A,B). The increase was greatest at right occipital and parietal channels and was absent from the analysis of non-phase-locked activity (see Figure 4). Cluster based permutation analysis demonstrated that it was not significantly different between standard and deviant conditions (demonstrated by the absence of significant differences in the 75–175 ms interval in Figure 3C), suggesting that it was a counterpart in the time-frequency domain of the P1 and N1 VEPs. A prominent reduction in overall power in a broad range of approximately 6–24 Hz was found at latencies 150–600 ms and was strongest at right occipital and parietal channels; the reduction was more pronounced for deviants than for standards. The same reduction was observed in non-phase-locked power analysis, suggesting it was non-phase locked in origin, and will be discussed below.

### Changes in non-phase-locked spectral power

A decrease in non-phase-locked spectral power, greatest at 14 Hz, was observed to both standard and deviant stimuli, see Figures 4A,B. The decrease, strongest at right occipital and parietal channels, began at 150 ms and lasted until approximately 525 ms in the standard condition and 600 ms in the deviant. (The exact timings and frequency ranges vary slightly from electrode to electrode.) Cluster based permutation testing demonstrated that the decrease in alpha spectral power was significantly stronger for deviant than for standard stimuli in the time interval between approximately 450 and 600 ms (Monte Carlo *p* = 0.0059), see Figures 4C and 2C. It should be noted that when the number of standard trials was matched to the deviant trials the stronger decrease in alpha spectral power to deviant stimuli was maintained. As previously highlighted, this alpha power decrease was also present in the analysis of the overall spectral power (see Figure 3), however its maintenance in the present analysis demonstrates that it largely resulted from non-phase locked oscillatory change.

### Inter trial phase locking

**Figure 5:**
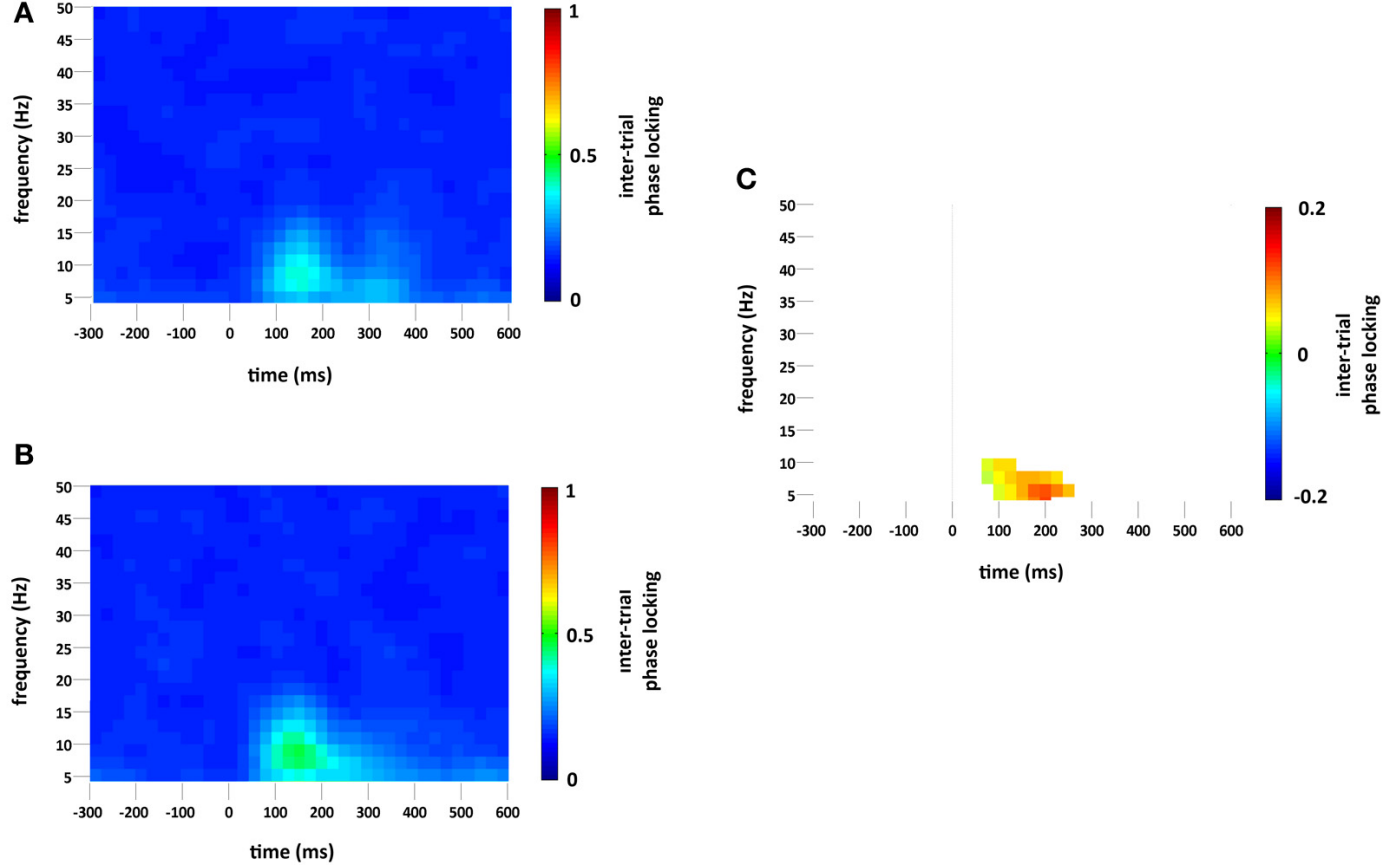
**ITPL (ranging from 0 to 1, with values approaching 1 indicating highly correlated phase values) at the occipital region of interest compared to baseline (−300 to 0 ms) for (A) standard and (B) deviant stimuli.** Plot **(C)** significant difference values (i.e., deviant minus standard, *p* < 0.05) after Monte Carlo permutation correction for multiple comparisons. Non-significant differences are masked in white.

Standard and deviant stimuli showed increased ITPL, greatest at 6–8 Hz, between approximately 75 and 350 ms, see Figures 5A,B. The increase in ITPL was broadly distributed across the scalp, although still strongest at occipital and parietal electrode sites. Cluster based permutation testing demonstrated that deviant stimuli elicited a significantly greater increase in ITPL compared to standard stimuli between 75 and 225 ms (Monte Carlo *p* = 0.0004, Figures 5C and 2D).

### Frequency band analysis

In order to enable a direct comparison between the current study and previous studies which performed time-frequency analyses on separate, conventionally-defined frequency bands, we also conducted our statistical analyses for the following *averaged* frequency bands: theta (4–8 Hz), alpha (8–14 Hz), beta (14–30 Hz), and gamma (30–50 Hz). As in the main analysis, cluster based permutation tests of spectral power change and ITPL were also calculated for the 0–600 ms interval compared to baseline (−300 to 0 ms), and only clusters that survived 10,000 Monte Carlo permutations are displayed. Observations from the main cluster analyses were replicated. Specifically, a decrease in overall and non-phase locked spectral power in the alpha band was larger to deviants than standards in the 400–600 ms time interval (Overall—Monte Carlo *p* = 0.003, Non-phase locked–Monte Carlo *p* = 0.004). A similar spatial distribution was observed to that in the main analyses, i.e., strongest at right occipital and parietal electrode sites. There were no other significant either overall or non-phase locked power changes in any other frequency band. ITPL was larger to deviant stimuli as compared to standard stimuli in 0–250 ms interval in the theta band only (Monte Carlo *p* = 0.001). A similar spatial distribution was observed to that in the main cluster analysis, i.e., broadly distributed across the scalp, although strongest at occipital and parietal electrode sites.

## Discussion

Both standard and deviant stimuli elicited large and equivalent increases in overall (i.e., phase-locked and non-phase-locked) theta power between 75 and 175 ms. The timing of this spectral change in the theta range strongly suggests it was a spectral counterpart of the P1-N1 complex in the VEP. In the simplest case, if the P1-N1 peaks were simply two peaks in a continuous sinusoidal wave, the frequency of that wave would be in the theta/low alpha range, an explanation also proposed by Klimesch et al. (2004).

vMMN was found to be associated with an increase in ITPL in the theta range, peaking at 6–8 Hz, which coincided with the early vMMN epoch observed in the VEP grand average waveform (133–263 ms). Hence, theta phase locking appears to play a role in the generation of the vMMN response, as it does in the aMMN response. This was followed by a significant decrease in non-phase-locked spectral power in the high alpha range, peaking at 14 Hz, which coincided with the late vMMN epoch (377–584 ms). These alpha-range oscillatory changes were strongest at right hemisphere occipital and parietal electrode sites.

The increase in theta ITPL in response to deviant stimuli was not accompanied by a significant increase in theta power, pointing toward a striking similarity between vMMN and aMMN generation. Recall that Fuentemilla et al. (2008) study reported that temporal subcomponent of the aMMN (generated by sources of aMMN in the auditory cortex) was driven by theta phase realignment without concurrent spectral power modulation. Similarly, in our study the early phase of the vMMN was purely a product of theta phase realignment that was not accompanied by theta power increase. The theta realignment effect had a broad scalp distribution but was most pronounced in the occipital sites, consistent with the idea of primary vMMN generators in the visual cortex plus additional generators in the frontal cortex (see below). More generally, theta phase locking (with or without concurrent theta power increase) was found in all other previous studies of aMMN (see Introduction).

Cumulatively the vMMN and aMMN findings point toward an important role for phase locking in the underlying mechanisms of the auditory and visual MMN. Phase locking has been suggested to play a key role in the linking of spatially disparate areas together into transitory neural networks. For example, theta and gamma phase locking between medial temporal lobe and hippocampal structures has been associated with successful memory formation (Fell et al., 2001; Rizzuto et al., 2003). Visual working memory performance has also been associated with increased beta phase locking within separate areas of the extra-striate cortex (Tallon-Baudry et al., 2001) and increased theta phase coherence (i.e., increased phase locking between two sites rather than within one) between prefrontal and posterior areas (Sarnthein et al., 1998). The aMMN network is thought to be comprised of bilateral temporal sources located in the auditory cortex with an additional frontal source(s) (Giard et al., 1991; Deouell, 2007), with theta phase locking proposed as a possible mechanism for the functional connection of these areas (Fuentemilla et al., 2008). Significant theta phase locking was also observed in frontal electrode sites as well as occipital and parietal sites in the current study, a similar pattern to that observed in the aMMN studies. There have been no studies to date examining the possibility of a frontal source in the vMMN response, however the prefrontal cortex has been suggested to form a key part of the feedback loop in Kimura's predictive coding model of the vMMN response (Kimura, 2012). Given that vMMN can also result in attentional orientation similar to the aMMN, for which the frontal source is suggested to be primarily responsible, the investigation of the existence of a frontal vMMN source is an interesting avenue for future work.

The decrease in spectral power between 8 and 20 Hz (spanning two classic oscillatory frequency bands, alpha, 8–14 Hz and beta, 14–30 Hz) was observed in both standard and deviant responses from 200 ms post-stimulus. This decrease peaked at 12–14 Hz (often known as the “high alpha” range) and was significantly stronger in the deviants than standards in the interval between approximately 375–575 ms. In previous research, alpha oscillations have often been examined by averaging the spectral power in a narrow pre-selected band around 10 Hz, e.g., 8–12 Hz. This, however, does not take into account the variation in peak alpha power across individuals, something that has been shown to vary considerably across individuals and age groups (Doppelmayr et al., 1998). The variation in individual peak alpha power can mean that upper alpha ranges can extend up to 15 Hz, i.e., into what is classically thought of as the beta range (Hanslmayr et al., 2005). In the main analysis of the current study we avoided the shortcomings resulting from “banding” a continuous frequency range and calculated spectral power using complex demodulation with a sampling step of 2 Hz across 4–50 Hz range. The spectral resolution of 2 Hz has a disadvantage of blurring some distinctions, e.g., 13 Hz oscillation likely to appear as both 12 and 14 Hz activity. Yet, it is our view that by not pre-selecting a narrow range around 10 Hz to explore alpha oscillations we have avoided the issue of fuzziness of the band boundaries and are subsequently able to obtain a more sensitive measure of event related spectral change.

Reductions in post-stimulus alpha power (or “alpha desynchronization”) are considered to be reflective of increased activation within a cortical region, and, vice versa, increases in power reflective of decreased activation. For example, in a visual task alpha oscillations increase in sensorimotor regions and reduce in occipital regions, whereas during a motor task the opposite pattern occurs (Pfurtscheller, 1992). Previous EEG studies of visual processing found reduction of non-phase-locked alpha power with an occipital locus approximately 450–600 ms post-visual stimulus (e.g., Müller and Keil, 2002; Gazzaley et al., 2008). In particular, alpha power reduction reported by Gazzaley et al. (2008) closely matched both the timing (approximately 400–600 ms post-visual stimulus) and broad spectral profile (i.e., peaking within the alpha range but extending into the beta range) of that observed in the current study.

Furthermore, the reduction in alpha power, although found for both standards and deviants, was stronger with deviant stimuli, corroborating the link between alpha desynchronization and task-specific effects (Klimesch et al., 1997). Gazzaley et al. (2008) found that reduction in alpha power was stronger for task-relevant than task-irrelevant visual stimuli. Müller and Keil (2002) used a feature-based attention paradigm and found significantly stronger alpha desynchronization in upper alpha power for non-targets that contained the attended feature (e.g., green color) than those that did not. They suggested that the effect may have been caused by an initiation (although not execution) of a motor response for non-targets that contained the attended feature. Similarly, in our study deviants represent the rare stimuli which activate change detection mechanisms and thus can be regarded as more task-relevant than standards, i.e., deviants require more inhibition than standards. In particular, as the change-detection mechanism underlying MMN plays an important role in the orientation of attention to novel or unexpected events, it may be that the increased alpha desynchronization with deviants reflects in part the shifting of attentional resources following a detection of change, represented by the earlier increase in theta phase locking. Finally, it may also be worth noting that the alpha suppression has not been observed in the studies of aMMN, hinting at the possibility of different functional value of alpha oscillations in visual vs. auditory modality (cf. Hsiao et al., 2009 for increased alpha power post-auditory stimulus in an oddball paradigm). Further investigation of the oscillatory activity associated with the vMMN response may help test and develop these hypotheses.

In sum, the vMMN response has distinct oscillatory characteristics that are typically lost in the averaging process used to measure VEPs. Theta phase locking is associated with the early vMMN epoch, and may reflect the temporary functional connection of the cortical areas involved in the vMMN response. It also suggests a common oscillatory mechanism behind the aMMN and vMMN responses. The examination of oscillatory changes alongside grand average VEP waveforms provides a more complete picture of the event related neural changes in the vMMN response.

### Conflict of interest statement

The authors declare that the research was conducted in the absence of any commercial or financial relationships that could be construed as a potential conflict of interest.

## Supplementary material

Supplementary Figure A **Sixty-four channel grand average plots for all time-frequency analyses for (A) standard, (B) deviant and (C) significant difference *t*-values (i.e., deviant minus standard, *p* < 0.05) after Monte Carlo permutation correction for multiple comparisons**. Non-significant differences are masked in white. X and Y axes scales for all electrode plots are indicated by the blank plot at the bottom of the figure. It should be noted that statistical data is based on the cluster based permutation analyses across all channels, timepoints and frequencies, i.e., controlling for multiple comparisons across spatial/temporal/spectral dimensions. Electrodes are presented in the plots individually in order to show the presence and absence of effects at each electrode in a more detailed manner than using scalp maps.
