# Supplementary figures and images for: Oscillatory characteristics of the visual mismatch negativity: what evoked potentials aren't telling us
Source: Front Hum Neurosci. 2013 Aug 1;7:426. doi: 10.3389/fnhum.2013.00426 (PMC3729976; doi:10.3389/fnhum.2013.00426)

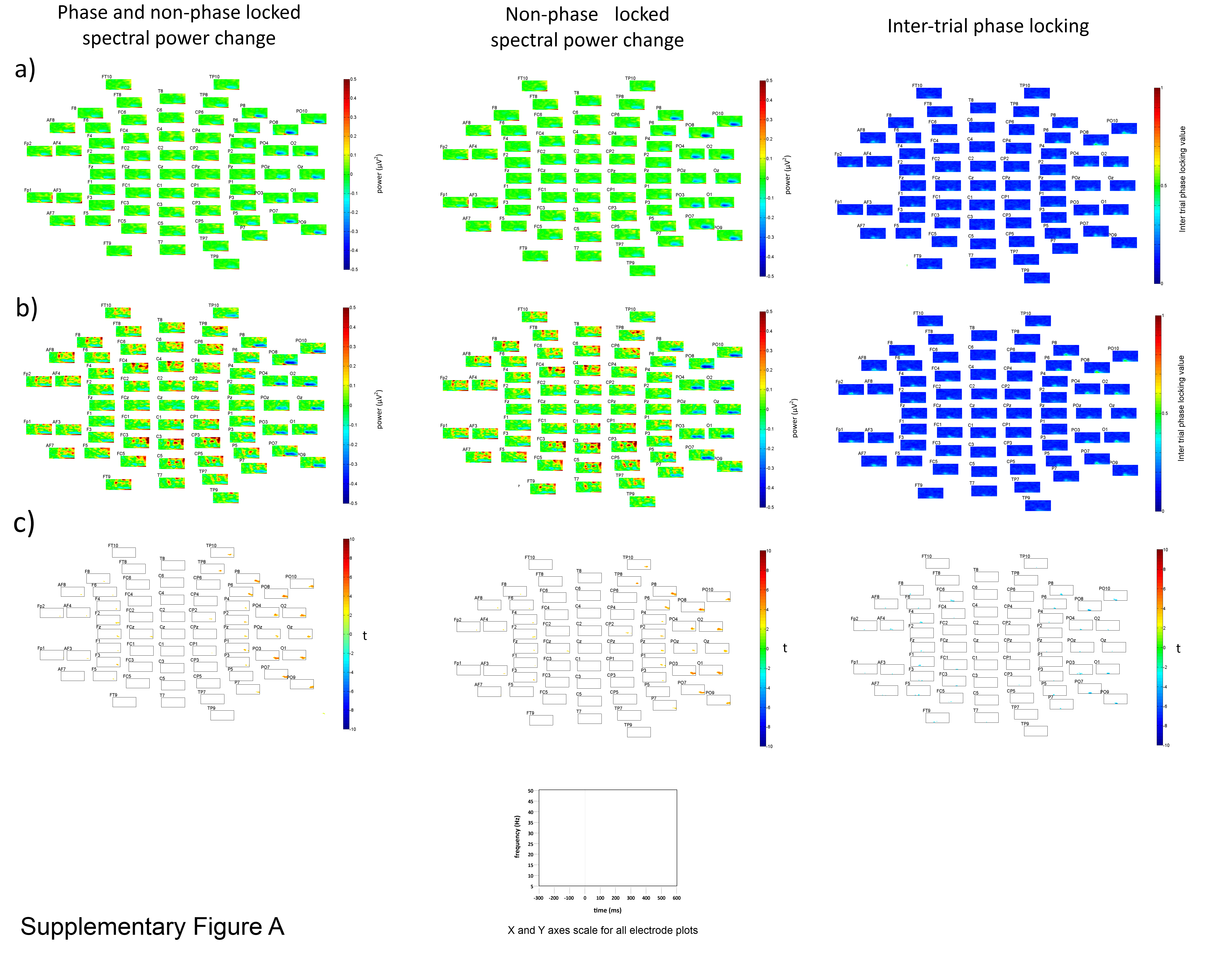

Supplement: Supplementary Figure A — Sixty-four channel grand average plots for all time-frequency analyses for (A) standard, (B) deviant and (C) significant difference t-values (i.e., deviant minus standard, p < 0.05) after Monte Carlo permutation correction for multiple comparisons. Non-significant differences are masked in white. X and Y axes scales for all electrode plots are indicated by the blank plot at the bottom of the figure. It should be noted that statistical data is based on the cluster based permutation analyses across all channels, timepoints and frequencies, i.e., controlling for multiple comparisons across spatial/temporal/spectral dimensions. Electrodes are presented in the plots individually in order to show the presence and absence of effects at each electrode in a more detailed manner than using scalp maps. [file Presentation1.ZIP › Supplementary figure A.tif]
